# Long-term follow up of single-chamber atrial pacing—system upgrade and Wenckebach block point behavior: potential implications for leadless AAI pacing?

**DOI:** 10.1007/s10840-025-02061-4

**Published:** 2025-05-16

**Authors:** Patrick Badertscher, Rebecca Arnet, Corinne Isenegger, Behnam Subin, Sven Knecht, Jessica Trussardi, Philipp Krisai, Felix Mahfoud, Christian Sticherling, Beat Schär, Michael Kühne

**Affiliations:** 1 Department of Cardiology, University Hospital Basel, Petersgraben 4, 4031 Basel, Switzerland; 2 Cardiovascular Research Institute Basel, University Hospital Basel, Basel, Switzerland

**Keywords:** Pacemaker, Sick sinus syndrome, AAI-pacing, Leadless pacing

## Abstract

**Background:**

Single chamber atrial pacing (AAI) provides a disease-specific treatment for sick sinus syndrome (SSS) but has largely been replaced by DDD pacing. With the advent of leadless atrial pacemakers (PM), there is growing interest in long-term follow-up data in patients with SSS and an AAI pacemaker.

**Purpose:**

To assess the incidence of system upgrade in patients treated with AAI-PM for SSS during long-term follow-up.

**Methods:**

This is an analysis of prospectively enrolled patients undergoing implantation of an AAI-PM. Wenckebach block point (WBP) was measured at implantation and serially during follow up.

**Results:**

We included 178 patients (58% female, median age at implantation 77 [71–83] years). The median follow-up duration was 6.5 [2.0–9.7] years. Twenty-three patients (13%) received a system upgrade to a DDD system, corresponding to a yearly upgrade rate of 2.0%. Median time to system upgrade was 5.2 [1.6–8.7] years. Reasons for system upgrade were higher-degree AVB (39%), atrial arrhythmias (35%), low WBP (17%), and syncope (9%). Median WBP at implantation was 130 [120–140] bpm, showing a significant decline over time in the upgrade-group compared to the rest of the cohort with 103 [91–130] bpm vs. 130 [120–130] bpm (*p* = 0.011).

**Conclusion:**

In this cohort of patients undergoing AAI-PM implantation for SSS, upgrade to a DDD system was low during long-term follow-up. Therefore, AAI pacing for the treatment of SSS may be considered a patient-tailored treatment option, especially in light of novel leadless pacing therapies.

**Graphical abstract:**

Central Illustration: AF = atrial fibrillation; AT = atrial tachycardia; AVB = atrioventricular block; WBP = Wenckebach block point; AAI = single-chamber atrial stimulation

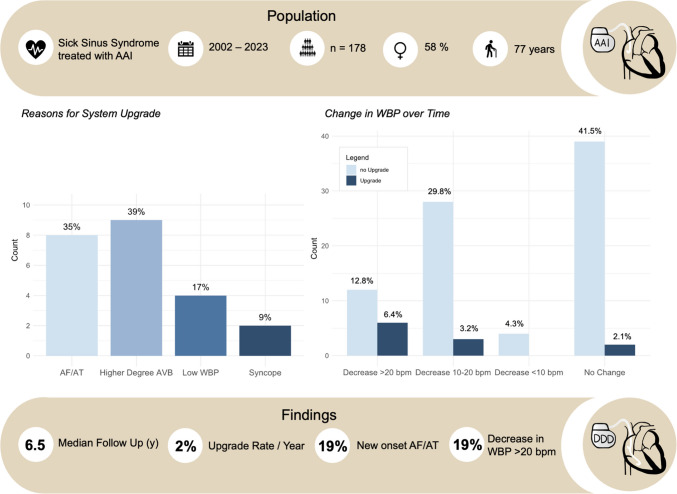

**Supplementary Information:**

The online version contains supplementary material available at 10.1007/s10840-025-02061-4.

## Introduction

The selection of the pacemaker (PM) mode for the management of sick-sinus syndrome (SSS) has been a protracted subject of deliberation. Historically, SSS was mainly treated with AAI pacing, as this system was considered disease-specific and more physiological compared to VVI and DDD and one RCT even suggested a lower rate of paroxysmal atrial fibrillation (AF) in AAI than in DDD pacing [1–10]. Additionally, AAI offered advantages over DDD pacing: a simpler and less time-consuming implantation process, fewer complications such as myocardial perforations, venous occlusions, or lead dislocations, higher cost-effectiveness due to the simplicity of the AAI system, and longer battery life [11–13].

With the DANish Multicenter Randomized Trial on Single Lead Atrial PACing vs. Dual Chamber Pacing in Sick Sinus Syndrome (DANPACE) trial, DDD pacing became the preferred pacing mode for SSS [14]. Although among 1415 patients no discernible disparities manifested in terms of all-cause mortality between AAI and DDD pacing, the yearly upgrade rate from AAI to DDD pacing was 1.7% [15]. Importantly, no differentiation between upgrades from AAI to DDD pacing due to incident atrioventricular (AV) block and changes performed as part of an elective generator exchange was made. Contrary to prior observations, DANPACE reported a higher occurrence of AF with AAI compared to DDD pacing.

AAI pacing currently has an indefinite class of recommendation in the ESC guidelines but is mentioned as an alternative if there are reasons to avoid two leads [14]. With the advent of a leadless pacemaker system for atrial pacing and in the era of widely available AF ablation, the choice of a single chamber system for patients with SSS may gain increased attention. Thus, long-term follow-up data of AAI-PM from large cohorts are needed to help identify patient groups potentially suited for AAI pacing. In addition, little is known about the role of serial Wenckebach block point (WBP) measurements in AAI-PM patients for monitoring and risk stratification.

The aim of this study was to assess the incidence of system upgrade of patients treated with AAI-PM for SSS during long-term follow-up and to investigate the role of serial WBP measurements for risk stratification.

## Methods

### Study design and patients

This is a retrospective analysis of prospectively enrolled patients undergoing implantation of an AAI-PM due to SSS at the University Hospital Basel. Exclusion criteria were patients treated with pacemaker systems other than AAI. The study was approved by the local ethics committees and adhered to the Helsinki Declaration.

### Implantation of AAI-PM and leads

PM implantation was performed in a standardized fashion using either a transvenous axillary approach or a cephalic cut-down. The atrial lead was positioned under fluoroscopic guidance. Only active fixation leads were used.

### Follow up protocol

Device interrogations were performed the day after implantation, 3 months after implantation and thereafter annually. Serial WBP measurements were obtained. Measurements of WBP directly before implantation until the day after implantation were regarded as baseline WBP. For the assessment of the WBP behavior over time, only patients with WBP measurement at implantation and at least one WBP measurement during follow-up were considered.

### System upgrade

System upgrade was performed in case of higher-degree AVB. In case of the occurrence of symptomatic atrial arrhythmias such as AF/atrial flutter (AFl) or atrial tachycardia (AT), the decision whether to perform a system upgrade for atrial arrhythmia management was at the discretion of the treating physician. Mode changes from AAI to VVI were counted as system upgrades since they required the implantation of an additional lead. Device explantations due to infection are reported separately.

### Study outcomes

The primary endpoint of this study was the rate of system upgrades in patients with an implanted AAI-PM. Secondary endpoints included the assessment of the WBP behavior over time as a predictor for the necessity of system upgrade and the occurrence of new onset AF/AT after AAI implantation.

### Statistics

Continuous variables were described using median with interquartile range (IQR) and were compared by Wilcoxon rank sum test. The absolute number of categorical variables was compared by Fisher’s exact test and Pearson’s chi-squared test, as appropriate. A *p*-value < 0.05 was considered statistically significant. Receiver-operating-characteristic (ROC) curves were constructed to assess the diagnostic accuracy of the absolute WBP and the delta WBP (ΔWBP) to predict the need for system upgrade during follow-up. The Youden Index was used to assess the maximal potential effectiveness of the WBP, thereby suggesting a cut-off with maximal sensitivity and specificity for the prediction of a system upgrade. A univariate regression was performed to assess possible predictive factors for system upgrade. All statistical analyses were performed using R (R Core Team (2021), R Foundation for Statistical Computing, Vienna, Austria) and RStudio 2023.09.1 RStudio Team (2019). RStudio, Inc., Boston, MA, USA.

## Results

### Implantation of AAI

We analyzed 178 patients with a median age of 77 [71–83] years undergoing AAI PM implantation between October 2002 and December 2023 at the University Hospital of Basel. One hundred three (58%) patients were female. Among all PM implantations at the University Hospital Basel during this time frame, an estimated 3.2% per year were AAI PM [16]. The following devices were implanted: 34% MicroPort, 26% Biotronik, 22% Medtronic, 16% Boston Scientific, and 2% Abbott.

Indications for AAI-PM implantation were sinus node dysfunction with tachy-brady-syndrome in 52 patients (29%), symptomatic sinus pauses or sinus arrest in 52 (29%), sinus bradycardia in 47 (26%), and chronotropic incompetence in 17 patients (10%). In 10 patients (6%), the indication was SSS but not further specified. Fifty-nine patients (33%) experienced dizziness (including vertigo and presyncope), 55 patients (31%) had syncope, and in 64 patients (36%) the symptoms were not further elucidated.

Median QRS duration was 98 [88–109] ms. 5 patients (3%) presented with right bundle branch block (RBBB) and 1 patient (1%) with left bundle branch block (LBBB). Five patients (3%) had left anterior fascicular block (LAFB). The median PR interval was 174 [162–204] ms. Thirty-four patients (19%) presented with AVB I° (Table 1).

**Table 1:** Baseline characteristics compared between the upgrade group and no-upgrade group

Baseline characteristics	Overall, *N* = 178	Upgrade, *N* = 23	No upgrade, *N* = 155	*p*-value
Sex (female)	103 (58%)	15 (65%)	88 (57%)	0.444
Age at implantation	77 [71–83]	75 [68–79]	78 [72–84]	0.054
Reasons for AAI implantation				0.120
Tachy-Brady-syndrome	52 (29%)	10 (43%)	42 (27%)	
Sinus pause/sinus arrest	52 (29%)	8 (35%)	44 (28%)	
Sinus bradycardia	47 (26%)	2 (9%)	45 (29%)	
Chronotropic incompetence	17 (10%)	1 (4%)	16 (10%)	
Not further specified	10 (6%)	2 (9%)	8 (5%)	
Symptoms before AAI implantation				0.292
Dizziness	59 (33%)	10 (43%)	49 (32%)	
Syncope	55 (31%)	8 (35%)	47 (30%)	
Other	64 (36%)	5 (22%)	59 (38%)	
QRS duration	98 [88–109]	110 [90–116]	96 [88–106]	0.198
RBBB	5 (3%)	0 (0%)	5 (3%)	> 0.999
LBBB	1 (1%)	1 (4%)	0 (0%)	0.129
LAFB	5 (3%)	0 (0%)	5 (3%)	> 0.999
PQ interval	174 [162–204]	164 [149–180]	176 [164–205]	0.091
AV block I°	34 (19%)	2 (9%)	32 (21%)	0.257
Atrial arrhythmia				0.166
None	76 (43%)	6 (26%)	70 (45%)	
Pre-existing	68 (38%)	10 (43%)	58 (37%)	
New-onset after AAI-implantation	34 (19%)	7 (30%)	27 (17%)	
Death	81 (46%)	7 (30%)	74 (48%)	0.120
Cardiovascular	15 (19%)	2 (29%)	13 (18%)	
Not cardiovascular	12 (15%)	2 (29%)	10 (14%)	
Unknown	54 (67%)	3 (43%)	51 (69%)	
WBP at implantation (bpm)	130 [120–140]	130 [128–140]	130 [120–140]	0.848
WBP at implantation (ms)	462 [500–429]	462 [469–429]	462 [500–429]	
WBP at last follow up (bpm)	128 [120–130]	103 [91–130]	130 [120–130]	0.011
WBP at last follow up (ms)	469 [500–462]	583 [659–462]	462 [500–462]	
ΔWPB (bpm)	− 10 [− 20–0]	− 25 [− 38 to − 15]	− 5 [− 20–0]	0.004

*p*-values were calculated using Pearson’s chi-squared test, Wilcoxon rank sum test, and Fisher’s exact test as appropriate. Continuous variables are described using median with interquartile range (IQR)

*RBBB* right bundle branch block, *LBBB* left bundle branch block, *LAFB* left anterior fascicular block, *WBP* Wenckebach block point

### System upgrade

**Fig. 1:**
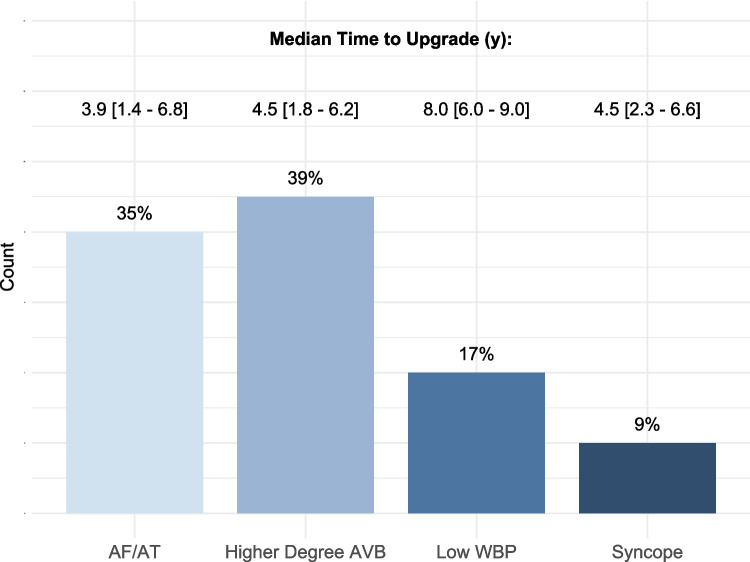
Reasons for system upgrade during follow up. AF = atrial fibrillation; AT = atrial tachycardia; AVB = atrioventricular block; WBP = Wenckebach block point

The median follow-up duration was 6.5 [2.0–9.7] years. In 155 patients (87%), a follow-up of at least 12 months was available. Twenty-four percent of all patients had a follow up of more than 10 years, 6% of more than 15 years, and 1% of more than 20 years. Twenty patients (11%) received a generator change after a median of 9.5 [8.3–11] years. During long-term follow-up, 23 patients (13%) received a system upgrade to DDD including 3 patients with a mode change to VVI only. In 4/23 cases (17%), the upgrade involved an occluded venous access, necessitating the use of a contralateral access in 3 patients, while in 1 case, an ipsilateral access was successfully achieved through dilation. The annual upgrade rate was 2.0%. Reasons for system upgrade were higher-degree AVB in 9 cases (39%), AF/AT in 8 cases (35%), low WBP in 4 (17%), and syncope despite AAI pacing in 2 patients (9%). Three out of 4 system upgrades due to low WBP were performed in patients meeting elective replacement indicator (ERI) criteria. None of these 4 patients had a PR interval at baseline of > 200 ms. One additional upgrade to DDD (0.6%) was performed after system infection with extraction of the AAI system. Among the 6 patients with RBBB and LBBB at baseline, 1 patient with LBBB received an upgrade during follow-up. Among patients undergoing system upgrade, 15 patients (65%) were female, and the median age was 80 [73–85] years. Median time to system upgrade was 5.2 [1.6–8.7] years (Fig. 1). The median ventricular pacing burden in patients undergoing system upgrade was 16 [1.4–95.0] % during follow-up after the upgrade.

### Wenckebach block point

**Fig. 2:**
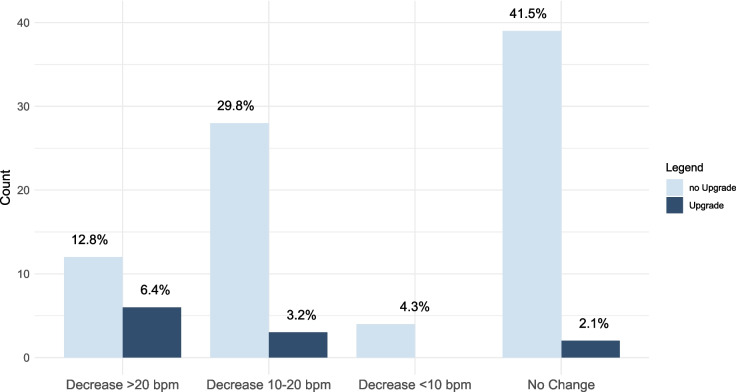
Changes in Wenckebach block point during follow up for patients without upgrade compared to patients with system upgrade

WBP at implantation was 130 [120–140] bpm overall. During a median follow up of 1698 days, 18 patients (19%) showed a reduction of WBP > 20 bpm, 31 (33%) a reduction of 10–20 bpm, 4 (4%) a reduction of < 10 bpm, in 41 (44%) the WBP remained stable (Fig. 2). When comparing patients with a system upgrade vs. patients with no system upgrade during long-term follow-up, the WBP at baseline was similar with 130 [128–140] bpm vs. 130 [120–140] bpm respectively (*p* = 0.848), and 99% of patients had a WBP > 100 bpm at the last available follow-up. However, the WBP was significantly lower at the last available device interrogation in patients receiving a system upgrade compared to patients with no system upgrade 103 [91–130] bpm vs. 130 [120–130] bpm, *p* = 0.011, respectively. The ΔWPB from baseline to last available WBP was − 25 [(− 38) to (− 15)] bpm in patients with system upgrade compared to − 5 [(− 20)–0] bpm in patients with no system upgrade. A reduction in WBP > 20 bpm was associated to a system upgrade with a specificity of 86% and a positive predictive value of 33%. The Youden Index, or cut-off value of maximal potential effectiveness, resulted in 106 bpm for the absolute WBP with a sensitivity of 58% and a specificity of 88%. The Youden Index of the ΔWBP resulted in a decrease of 17.5 bpm with a sensitivity of 73% and a specificity of 71%. The area under the curve (AUC) from the ROC for the absolute WBP was 0.72. The AUC from the ROC for the ΔWBP was 0.77.

### Atrial arrhythmias

Atrial arrhythmias were present in 68 patients (38%) at the time of implantation. Fifty-eight patients (33%) had a history of paroxysmal AF and 10 patients (18%) of AFl/AT. Among these 68 patients, 10 patients (15%) received a system upgrade during follow-up. Overall, 34 patients (19%) developed new-onset atrial arrhythmias after Implantation, of which 7 patients (21%) underwent a system upgrade during follow-up. Of all patients receiving a system upgrade because of AF (*n* = 8), 7 had developed new-onset atrial arrhythmias (3.9%) and only 1 patient had pre-existing atrial arrhythmias (0.6%).

### Medical therapy

Among the 23 patients (13%) receiving a system upgrade, 19 patients (83%) received betablocker or calcium channel blocker therapy and 7 patients (30%) additionally received antiarrhythmic drug therapy with amiodaron or flecainide. Among the reasons for system upgrade, medical rate or rhythm control was present in 6 (67%), 7 (88%), 4 (100%), and 2 (100%) cases for patients with higher-degree AVB, atrial arrhythmia, low WBP, and patients suffering from syncope despite prior AAI implantation, respectively.

### Predictors for system upgrade

When performing a univariate analysis for the prediction of system upgrade during follow-up, a decrease in WBP of > 20 bpm showed an odds ratio of 9.8 (*p* = 0.010), while neither age, gender, presence of atrial tachycardia/arrhythmia or WBP at implantation were predictive for the need of a system upgrade (Table 2).

**Table 2:** Univariate analysis for the prediction of system upgrade during follow up

Characteristic	*N*	OR	95% CI	*p*-value
Sex	178			
Male		0.70	0.27, 1.71	0.446
Age at AAI implantation	178	0.97	0.93, 1.01	0.149
Atrial arrhythmia	178			
None		—	—	
Pre-existing		2.01	0.70, 6.22	0.201
New-onset after AAI-implantation		3.02	0.93, 10.2	0.065
WBP at implantation	156	1.00	0.97, 1.03	0.864
ΔWBP	94			
No change		—	—	
Decrease > 20 bpm		9.75	1.96, 72.9	0.010
Decrease 10–20 bpm		2.09	0.33, 16.7	0.436
Decrease < 10 bpm		0.00		0.994

*OR* odds ratio, *CI* confidence interval, *WBP* Wenckeback block point

### Sex-specific differences

Among the 178 patients with AAI PM, 103 (58%) patients were female, and among the 23 patients (13%) receiving a system upgrade to DDD during long-term follow-up, 15 (65%) patients were female. No significant sex-specific differences were noted for WBP at implantation, WBP at last follow-up, or ΔWBP.

## Discussion

In this large cohort of patients undergoing AAI implantation for SSS, we report the following main findings:

First, over a follow-up of 6.5 years, 13% of all patients received a system upgrade, including mode changes to VVI. This corresponds to an annual upgrade rate of 2%. Second, while most system upgrades were performed for higher-degree AVB, symptomatic AF/AT represented the second most common cause. Third, system upgrades are difficult to predict based on clinical variables including the WBP at the time of device implantation. Fourth, ΔWPB from baseline to last available WBP was significantly lower in patients with system upgrade compared to patients with no system upgrade with (− 25 bpm vs. − 5 bpm). A decrease in WBP of > 20 bpm showed an odds ratio of 9.8 (*p* = 0.010) for the need of system upgrade. The AUC from the ROC for the ΔWBP was 0.77, thereby providing some guidance for risk stratification during follow-up in this patient population.

Our findings corroborate and extend findings in the current literature [1, 5, 6, 8–10, 15, 17–19]. A detailed overview of studies assessing AAI pacing is provided in Supplemental Table S1. The main reason for using DDD pacing in patients with SSS is to avoid the potential need of a system upgrade due to the need of ventricular pacing during follow-up. Yearly upgrade rates are inconsistently calculated in the literature: some studies used the whole follow-up duration, while others only used the follow-up duration of patients requiring a system upgrade [1, 5, 6, 8–10, 15, 17–19]. Thus, the annual upgrade rate in this study is 2%, when averaged over the median follow-up time of all patients (6.5 years) and 1.5% when averaged over the follow-up time of upgraded patients (8.9 years). Importantly, most prior studies did not differentiate between the requirement of a system upgrade due to incident AVB or prophylactic DDD upgrade during generator change [9, 10, 17]. Furthermore, most system upgrades were performed later during follow-up in prior studies. In this study, only one-third of patients receiving a system upgrade due to higher-degree AVB were performed in the first 2 years after implantation (Supplemental Table S2). Seventy-five percent of system upgrades due to low WBP were performed in patients meeting ERI criteria.

While there has been conflicting evidence regarding an increased risk of atrial arrhythmias in AAI pacing, in this study, 34 patients (19%) were found to have atrial arrhythmias during follow-up. Most upgrades due to AF/AT were performed between 2005 and 2017 in the current study. With the advent of catheter ablation therapy, it is likely that the rate for system upgrade due to atrial arrhythmias can be reduced in the future. The median ventricular pacing burden in patients undergoing system upgrade due to AF/AT was ≤ 5% in 71% of patients. Furthermore, since only 7/34 patients with atrial arrhythmia needed a system upgrade during follow-up, it is likely that the rate of subclinical AF/AT detected via CIED with unclear clinical consequences might also play a non-negligible role in this patient population.

The suitability of the WBP at implantation has been examined in various studies, yielding disparate results. Masomuto et al. identified a WBP < 120 bpm at implantation as a significant predictor for the development of higher-degree AVB [8]. Conversely, Tripp et al. found no significant difference in WBP at implantation between patients with and without upgrades [9]. There is little evidence on the behavior of the WBP over time in patients with AAI and SSS. Andersen et al. noted spontaneous fluctuations in WBP over time, likely attributable to physiological changes in autonomic tone, and found no differences in patients requiring a system upgrade compared to patients with no upgrade [5]. Our findings show that serial WBP measurements may provide valuable information regarding temporal dynamics of AV conduction. These results, however, should be considered hypothesis-generating to guide future studies.

With the introduction of a DDD leadless system, the “forgotten” AAI pacing mode might gain increased popularity in the near future [20]. It has been shown that the complication rates between transvenous and leadless PM system differ: Most common complications of transvenous systems include lead fracture, dislodgement, infection, venous occlusion, tricuspid regurgitation [8, 17, 21], while leadless systems might have a slightly higher risk of pericardial effusion [22, 23]. A system upgrade, in the rare case where this is necessary, might be more easily performed using a leadless system compared to a transvenous system, but this needs to be shown in future studies.

We note the following limitations: First, this is a retrospective analysis of prospectively enrolled patients in a single center study with all its limitations. Second, the impact of factors such as anesthesia or heart rate modulating medication on the WBP was not assessed. However, in this study, WBP was measured at implantation and throughout follow-up to track changes in AV conduction over time potentially counterbalancing temporary fluctuations. Third, conventional transvenous PM systems were assessed, thus our findings might not be translated in a 1:1 fashion to leadless systems. In addition, with only one company offering leadless atrial leads at present, the technology is still in its early stages.

In conclusion, in this cohort of patients undergoing AAI-PM implantation for SSS, system upgrade to a DDD system was rare (2% per year) during long-term follow-up. AAI pacing as a disease-specific therapy for the treatment of SSS may warrant increased consideration in light of novel treatment options for leadless pacing.

## Supplementary Information

Below is the link to the electronic supplementary material.

ESM 1 (DOCX 157 KB)

## Data Availability

The datasets generated during and/or analyzed during the current study are available from the corresponding author on reasonable request.
