# Supplementary material for: Long-term follow up of single-chamber atrial pacing—system upgrade and Wenckebach block point behavior: potential implications for leadless AAI pacing?
Source: J Interv Card Electrophysiol. 2025 May 16;68(8):1605–12. doi: 10.1007/s10840-025-02061-4 (PMC12476441; doi:10.1007/s10840-025-02061-4)
Supplement: Supplementary file 1 — (DOCX 157 KB) [file 10840_2025_2061_MOESM1_ESM.docx]

**Supplement**

**Supplement Table 1**

| **Publication (DOI)** | **Publication year** | **N =** | **Centers** | **Prospective (p) / retrospective (r)** | **RCT / observational (o)** | **Analyzed pacing modes** | **Yearly upgrade rate (%)** | **AVB (n)** | **AVB % per year** | **AF (n)** | **AF (%) per year** | **AF_AAI_ (%) / AF_DDD_ (%)** | **WBP over time** |
| --- | --- | --- | --- | --- | --- | --- | --- | --- | --- | --- | --- | --- | --- |
| [10.1161/01.cir.98.13.1315](https://doi.org/10.1161/01.cir.98.13.1315) | 1998 | 225 | single | p | RCT | AAI (110), VVI (115) | 0.6 | 4 | 0.6 | NA | NA | NA | yes |
| 10.1016/s0300-8932(02)76799-7 | 2002 | 160 | single | r | o | AAI (160) | 1.2 | 7 | 0.8 | 32 | 3.7 | 20 / - | no |
| 10.1046/ j.1460-9592.2001.00358.x | 2003 | 399 | single | r | o | AAI (399) | 2.5 | 20 | 1.1 | 10 | 0.6 | 2.5 / - | no |
| [10.1016/j.eupc.2004.05.003](https://doi.org/10.1016/j.eupc.2004.05.003) | 2004 | 196 | multi | r | o | AAI (95), DDD (101) | 1.3 | 8 | 1.1 | 4 | 0.5 | 4.2 / 7.9 | no |
| [10.1111/j.1540-8159.2005.08672.x](https://doi.org/10.1111/j.1540-8159.2005.08672.x) | 2005 | 117 | single | r | o | AAI (117) | 1.4 | 6 | 0.7 | 3 | 2.6 | 0.4 / - | no |
| [10.3349/ymj.2010.51.6.832](https://doi.org/10.3349/ymj.2010.51.6.832) | 2010 | 186 | single | r | o | AAI (73), DDD (113) | 0.9 | 4 | 0.9 | 2 | 0.5 | 2.8 / 15.2 | no |
| [10.1093/eurheartj/ehr022](https://doi.org/10.1093/eurheartj/ehr022) ***** | 2011 | 1415 | multi | p | RCT | AAI (707), DDD (708) | 1.7 | NA | NA | 201 | 5.3 | 28.4 / 23.0 | no |
| [10.5603/KP.a2014.0148](https://doi.org/10.5603/kp.a2014.0148) | 2015 | 809 | single | r | o | AAI (86), DDD (723) | 2.8 | 4 | 0.9 | 8 | 1.7 | 9.3 / 11.2 | no |
| [10.1093/europace/euw364](https://doi.org/10.1093/europace/euw364) ****** | 2017 | 1384 | multi | p | RCT | AAI (696), DDD (688) | 4.5 | NA | NA | 199 | 3.2 | 28.6 / 29.1 | no |
| 10.1016/j.ihj.2023.12.004 | 2024 | 113 | single | r | o | AAI (113) | 0.0 | 0 | 0 | 4 | 0.9 | 3.5 / - | no |

****** Long term follow-up of the * DANPACE publication in 2011 (DOI: [10.1093/eurheartj/ehr022](https://doi.org/10.1093/eurheartj/ehr022))

**Supplement Table 2**

| **Sex** | **Age (y)** | **Time to Upgrade (d)** | **Reason** | **ERI** |
| --- | --- | --- | --- | --- |
| m | 70 | 4786 | Complete AVB after mitral valve replacement | no |
| f | 66 | 645 | Paroxysmal complete AVB in the setting of accumulation of beta-blockers in severe renal insufficiency. | no |
| f | 81 | 2 | Unclear circumstances: syncope in the early morning with paroxysmal complete AVB. | no |
| f | 57 | 1657 | Paroxysmal AVB II° Type Mobitz with 2:1 conduction in the early morning with symptoms of dizziness | no |
| f | 58 | 825 | Multiple syncope with NSTEMI and reduced LVEF as well as one-time documented AVB III° | no |
| f | 87 | 1949 | Dizziness at rest and under stress with possible AVB | no |
| m | 77 | 3214 | Paroxysmal AVB II-III° at night | yes |
| f | 64 | 2275 | Higher-degree AVB under stress | no |
| m | 77 | 164 | Persisting AVB II° Type Mobitz with 2:1 conduction | no |
